# Dedifferentiated Chondrocytes in Composite Microfibers As Tool for Cartilage Repair

**DOI:** 10.3389/fbioe.2017.00035

**Published:** 2017-06-13

**Authors:** Marco Angelozzi, Letizia Penolazzi, Stefania Mazzitelli, Elisabetta Lambertini, Andrea Lolli, Roberta Piva, Claudio Nastruzzi

**Affiliations:** ^1^Department of Biomedical and Specialty Surgical Sciences, University of Ferrara, Ferrara, Italy; ^2^Department of Life Sciences and Biotechnology, University of Ferrara, Ferrara, Italy; ^3^Department of Orthopaedics, Erasmus MC, University Medical Center, Rotterdam, Netherlands

**Keywords:** microfibers, extracellular matrix-derived biomaterials, chondrocytes, cartilage tissue engineering, gene expression

## Abstract

Tissue engineering (TE) approaches using biomaterials have gain important roles in the regeneration of cartilage. This paper describes the production by microfluidics of alginate-based microfibers containing both extracellular matrix (ECM)-derived biomaterials and chondrocytes. As ECM components gelatin or decellularized urinary bladder matrix (UBM) were investigated. The effectiveness of the composite microfibers has been tested to modulate the behavior and redifferentiation of dedifferentiated chondrocytes. The complete redifferentiation, at the single-cell level, of the chondrocytes, without cell aggregate formation, was observed after 14 days of cell culture. Specific chondrogenic markers and high cellular secretory activity was observed in embedded cells. Notably, no sign of collagen type 10 deposition was determined. The obtained data suggest that dedifferentiated chondrocytes regain a functional chondrocyte phenotype when embedded in appropriate 3D scaffold based on alginate plus gelatin or UBM. The proposed scaffolds are indeed valuable to form a cellular microenvironment mimicking the *in vivo* ECM, opening the way to their use in cartilage TE.

## Introduction

The main component of the articular cartilage is an extracellular matrix (ECM) containing a relative small number of cells. The ECM is mainly composed of collagen type II and proteoglycans, determining the typical mechanical characteristics of cartilage (i.e., tensile strength, flexibility, and resistance to compressive loads) (Muir, 1995). Self-healing of damaged cartilage is unfortunately very limited, being chondrocytes unable to grow in the dense ECM. Nowadays, the cartilage reparative strategies, aiming to generate a functional tissue, are mainly based on the combined use of cells, scaffolds, and biomolecules (Poole et al., 2001; Chang et al., 2005; Moutos and Guilak, 2008; Liao et al., 2014).

Tissue engineering (TE) approaches for cartilage require some key factors: they include (a) an ideal cell source, (b) a precise control of cell differentiation (e.g., using soluble chemical factors and mechanical stimulation), and (c) an adequate scaffold based on specific biomaterials (Chang et al., 2005; Brown and Badylak, 2014).

With respect to the cells, chondrocytes being the cellular component of the mature cartilage are the most obvious choice for cartilage TE (Dell’Accio et al., 2001; Melero-Martin and Al-Rubeai, 2007). However, the use of chondrocytes is limited by rareness of the donor tissues, the instability in *in vitro* culture, and the loss of differentiated phenotype due to cell expansion. The maintenance of the chondrocytic phenotype is currently performed by 3D environments supplemented with chondrogenic inducers (i.e., TGFβ) (Khaghani et al., 2012). The approaches are mainly consisting of natural or synthetic scaffold offering a favorable milieu for chondrogenesis (Yang et al., 2008; Youngstrom et al., 2015).

Hydrogels, particularly those based on alginate, resulted successful in chondrocyte redifferentiation (Guo et al., 1989; Häuselmann et al., 1996; Caron et al., 2012). Alginates form indeed biocompatible, biodegradable, and shape-adaptable structures that are largely employed for cell embedding. Notably, alginate gels were proposed for different *in vivo* applications; they allow bidirectional exchange of nutrient, oxygen, and cell waste products, protecting at the same time the cells from the host immune system (Calafiore, 2003; Penolazzi et al., 2010; Mazzitelli et al., 2013; Bidarra et al., 2014). Alginate is particularly appealing for chondrocytes immobilization since it supports the phenotype maintenance as proved by the typical rounded morphology displayed by chondrocyte in alginate, sustaining the cartilage ECM production (Guo et al., 1989; Bonaventure et al., 1994; Häuselmann et al., 1996).

Despite many positive properties, alginate scaffolds are far from representing an environment strictly mimicking the biological ECM where chondrocytes reside, reach of various biochemical signals. Their lack affects the interaction between the entrapped/seeded cells and the biomaterial and compromises the onset of molecular signaling that guides the effective integration of the implanted construct with the surrounding host tissue (Lee and Mooney, 2001).

For possibly solving the limitations of alginate-based scaffolds, in this study, an improvement has been proposed, developing microfibrous alginate scaffold containing ECM components such as gelatin (a soluble, partially hydrolyzed, and collagen derivative) or the urinary bladder matrix (UBM) (a natural decellularized matrix, derived from porcine bladder).

These natural materials confer to the scaffold elements resembling the original ECM collagenous network and supporting cell adhesion, migration, and differentiation by the presence of glycosaminoglycans (GAGs) (Badylak et al., 2009; Gómez-Guillén et al., 2011; Santoro et al., 2014). Notably, UBM is one of the most representative decellularized materials that have received regulatory approval for use in human patients (Gilbert et al., 2006). It has been demonstrated that the presence and integrity of basement membrane complex in UBM promotes inductive tissue remodeling (Brown and Badylak, 2014), but little is known about the supporting activity of UBM toward chondrocyte function. UBM was recently used for articular cartilage regeneration in canine and murine models demonstrating its efficacy in treating dogs or mice with chronic osteoarthritis of the hip or knee joint, respectively (Rose et al., 2009; Tottey et al., 2011; Jacobs et al., 2017).

Particularly, composite microfibers (i.e., 3D scaffolds), potentially suitable for a fiber-based tissue such as cartilage, have been designed and produced by a specific microfluidic approach (Angelozzi et al., 2015).

Lab-on-a-chip (LOC) devices based on microfluidic chips have been recently proposed as miniaturized bioanalytical systems for chemical/biological applications being able to perform multiple tasks associated with many laboratory procedures. LOC devices offer indeed many advantages over standard (i.e., macroscopic) systems, including reduced sample and reagent consumption, faster analysis, and higher levels of throughput and automation. Despite these advantages, the production of biomaterial based scaffold by microfluidics has still limited example in the current literature.

As cellular component, human advanced dedifferentiated nasal chondrocytes from monolayer passage P6 were employed. Chondrocytes derived from the nasal septum are highly promising cell source for the repair of articular cartilage defects since a great capacity to generate hyaline-like cartilage tissues, with the plasticity to adapt to a joint environment has been demonstrated (Kafienah et al., 2002; Wolf et al., 2008; Mumme et al., 2016).

This paper describes the potential of composite microfibers with respect to their ability to control chondrocyte differentiation for proper cartilage matrix reconstruction.

The effect of microenvironment around individual mature chondrocytes in microfibers was also considered; it is well known indeed that chondrocytes in their natural environment are present as single cells with a spherical shape, surrounded by ECM not allowing for cell-to-cell contacts. The properties of the produced composite microfibers were investigated *in vitro* conditions excluding the presence of exogenously added chondrogenic inducers.

In addition, in view of a possible use of the composite microfibers as scaffold for cryopreservation, experiments were undertaken to evaluate their potential as banking for chondrocytes.

In this respect, the properties of the embedded chondrocytes after a freeze and thaw procedure have been investigated. The obtained results suggest that the microfibrous embedded chondrocytes could be employed to deliver the freshly thawed constructs at the operating theater.

## Materials and Methods

### Chondrocyte Cultures

Cartilage fragments from nasal septum were obtained from 15 donors between 25 and 60 years old, which underwent septoplasty surgery procedures, after informed consent and approval of the Ethics Committee of the University of Ferrara and S. Anna Hospital. Briefly, cartilage fragments were minced into small pieces and rapidly incubated with type VIII Collagenase (Sigma-Aldrich Chemical Co., St. Louis, MO, USA) at 37°C for 16 h (do Amaral et al., 2012). Cells were harvested by centrifugation and plated (p0) at a density of 20,000 cells/cm^2^ in tissue culture flasks (25 cm^2^) or 8-well culture slides in standard medium (50% DMEM high-glucose/50% DMEM F-12/10% fetal calf serum) (Euroclone S.p.A., Milan, Italy) supplemented with antibiotics (penicillin 100 mg/mL and streptomycin 10 mg/mL), at 37°C in a humidified atmosphere of 5% CO_2_. After 7 days, the culture medium was removed and then changed twice a week. At 70–80% confluence, cells were scraped off by 0.05% TRYPSIN EDTA (Gibco, Grand Island, NE, USA), washed, plated, and allowed to proliferate in standard conditions (50% DMEM high-glucose/50% DMEM F-12/10% fetal calf serum) to induce chondrocyte dedifferentiation (until p6). Dedifferentiated chondrocytes at different culture passages were scraped off counted by hemocytometric analysis, assayed for viability, and thereafter used for molecular analysis or for encapsulation procedures. During the *in vitro* expansion, dedifferentiated chondrocytes underwent a total of 19–21 population doublings.

### UBM Isolation and Purification

Porcine urinary bladders were harvested from pigs, immediately following euthanasia. The connective tissue excess and the residual urine were removed. By mechanical treatment, the tunica serosa, tunica muscularis externa, tunica submucosa, and majority of the tunica muscularis mucosa were removed; thereafter, urothelial cells of the tunica mucosa were detached from the luminal surface by incubating the tissue in saline solution. The resulting tissue, which was composed of the basement membrane of the urothelial cells plus lamina propria, is termed “urinary bladder matrix,” shortly UBM. The obtained UBM sheets were then treated by a solution containing 0.1% (v/v) peracetic acid (Sigma), 4.0% (v/v) ethanol (Sigma), and 95.9% (v/v) sterile water for 2 h. Peracetic acid residues were then removed with washes with phosphate-buffered saline (PBS, pH 7.4) and sterile water. The decellularized UBM was then lyophilized and milled to obtain a particulate form using a Wiley Mini Mill (Thomas Scientific, Swedesboro, NJ, USA).

### Microfiber Production and Encapsulation Procedure

Fluidic reagents were introduced into the microfluidic network from glass gastight syringes (Hamilton, Reno, NV, USA) by syringe pumps (model KD100, KD scientific Inc., Holliston, MA, USA).

**Figure 1:**
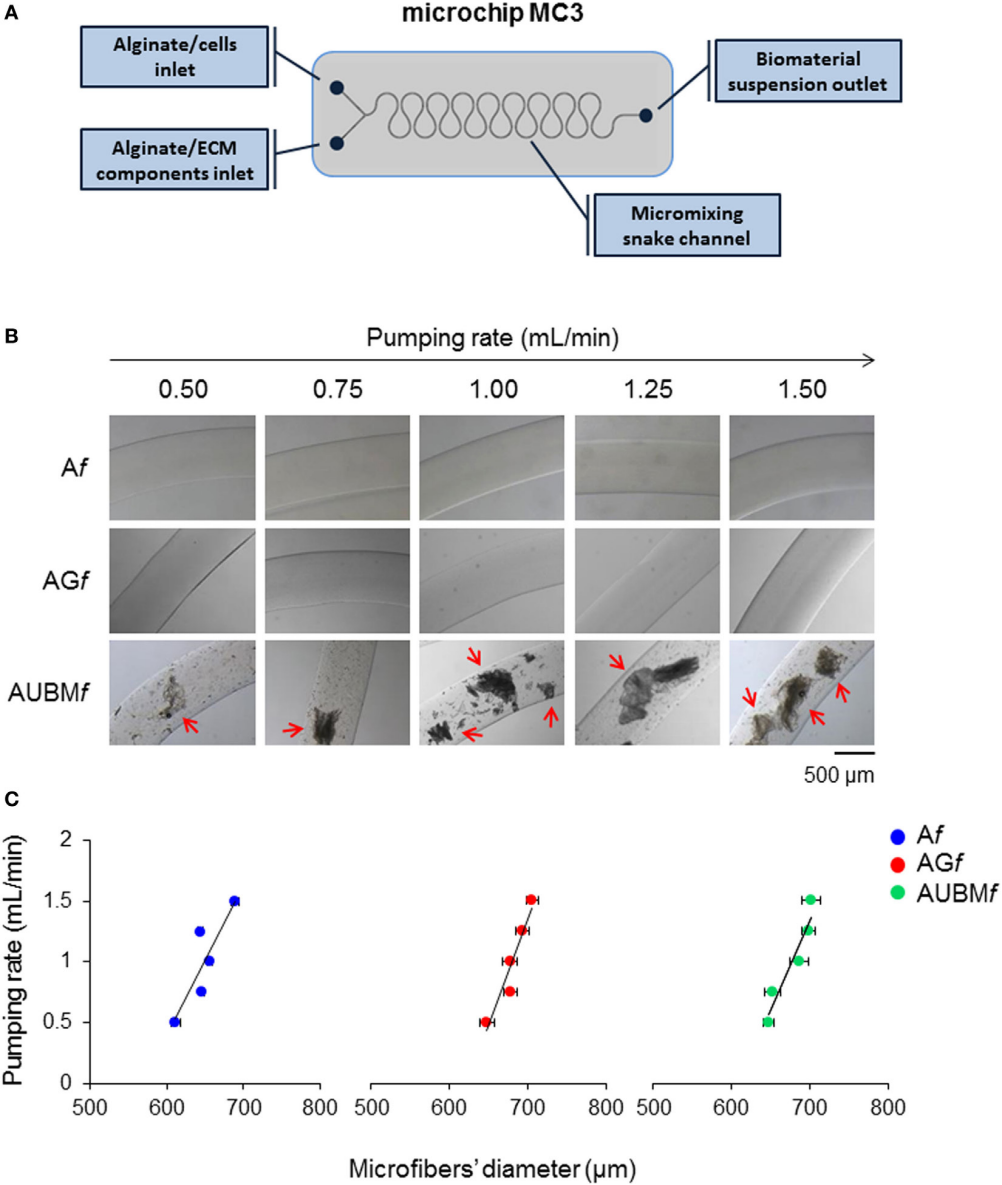
Production and characterization of composite microfibers. **(A)** Schematic representation of the snake micromixing microfluidic device employed for the production of composite microfibers. The device presents two inlets where the alginate suspension containing cells or gelatin or UBM were delivered through the micromixing snake geometry channel and a 700-µm outlet tube (#T3) into a BaCl_2_ solution in order to obtain alginate (A*f*), alginate plus gelatin (AG*f*), or alginate plus UBM (AUBM*f*) microfibers. **(B)** Photomicrographs of A*f*, AG*f*, and AUBM*f* obtained at different pumping rates (from 0.5 to 1.5 mL/min). Arrows indicate the presence in AUBM*f* of UBM particles, in form of flat flakes. Bar corresponds to 500 µm. **(C)** Effect of the pumping rate on the dimension of the produced microfibers.

“Empty” and “multifunctional” (containing cells, ECM components or both) barium alginate microfibers were produced with a snake micromixing microfluidic device (Figure 1A). A sodium alginate dispersion in water (1.5–2.5%, w/v), used as main constituent of the microfibers was introduced into one inlet of the microchip at appropriate flow rates (0.50–1.50 mL/min). Plain sodium alginate dispersion or sodium alginate suspensions were delivered *via* the second inlet. The suspensions contained different amounts (1.125–4.500% for gelatin or 0.1–1.0% for UBM) of either ECM components (i.e., gelatin or UBM) or cells (i.e., dedifferentiated chondrocytes at 2 × 10^6^ cells/mL). Notably, gelatin forms a homogeneous dispersion in the aqueous sodium alginate, whereas UBM, being unsoluble in water, forms a suspension. The output from the outlet of the chip was transferred *via* a Teflon tube, into a BaCl_2_ solution (6.0 mM in water) where the Na-alginate flow stream was gelled to produce the final Ba-alginate consolidated microfibers. The plain alginate, the alginate/gelatin, or alginate/UBM microfibers are named *Af, AGf*, and *AUBMf*, respectively. Cells-containing microfibers were washed three times with saline before culturing in standard chondrocyte medium at 37°C in a humidified atmosphere of 5% CO_2_.

### Geometrical and Morphological Analysis of Microfibers

The dimension and morphology of microfibers were evaluated by optical stereomicroscopy. Quantitative analyses were performed using the photomicrograph analysis software EclipseNet. The mean diameter of the microfibers (±SD) was obtained by taking nine measurements along the (10 mm) length of the samples at equal intervals, in triplicate. Additionally, the distribution of different amount of gelatin (1.125, 2.25, and 4.5% w/v) and UBM (0.1, 0.5, and 1% w/v) in composite microfibers was evaluated by Coomassie Brilliant Blue staining. For the staining, the microfibers were incubated in 0.1% Coomassie Brilliant Blue R-250, 50% methanol, and 10% glacial acetic acid at room temperature for 1 h and then washed overnight at room temperature in the destaining solution (40% methanol and 10% glacial acetic acid). Gelatin and UBM distribution was determined by optical stereomicroscopy.

### Micromass Culture System

Dedifferentiated chondrocytes from monolayer culture were harvested and resuspended in standard medium at 2 × 10^7^ cells/mL density. Droplets (10 µL) were carefully placed in a 12-well plate. Cells were allowed to adhere for 3 h, followed by the addition of 1 mL of standard medium supplemented with 10% FCS. After 24 h, the cell droplets coalesced and became spherical. Medium was changed every 3 days, and micromasses were harvested on days 7 and 14.

### Viability Analysis of Encapsulated Cells

Viability of encapsulated cells was assessed immediately after encapsulation procedure and at days 7 and 14 of culture. Cell viability was evaluated by Calcein-AM/propidium iodide (PI) staining (Cellstain double staining kit, Sigma-Aldrich).

Before staining, the medium was removed from the wells, and 500 µL of the staining solution was added to each well. The samples were incubated in the dark at room temperature for 15 min, thereafter the wells were rinsed with PBS and immediately imaged using epifluorescent microscopy. Viability was measured *via* cell counting and automated analysis using ImageJ. Samples were visualized under a fluorescence microscope (Nikon, Optiphot-2; Nikon Corporation, Tokyo, Japan) dead cells stained red, while viable ones appeared green (Penolazzi et al., 2012).

### Microfiber Dissolution and Cell Recovery

The cells embedded in microfibrous scaffolds were retrieved, after gel dissolution at days 7 and 14 of *in vitro* culture. Microfibers were dissolved by incubation at 37°C in a 100 mM EDTA solution (pH 7.0) for 10 min. Cell suspension after microfiber dissolution was filtered with a Falcon™ 70 µM Nylon Cell strainer (BD Biosciences, Franklin Lakes, NJ, USA) and employed for successive analyses.

### GAG and DNA Quantification

Dedifferentiated chondrocytes before encapsulation procedure or recovered chondrocytes from microfibers or micromasses were lysed with 50 µL of RIPA buffer. Total sulfated GAG content was determined in RIPA samples from days 7 and 14 cultures by using 1,9-dimethylmethylene blue (DMMB), as previously described (Caron et al., 2012). A standard curve of chondroitin sulfate in PBS-EDTA was included to determine the GAG concentration in the samples. About 100 µL of diluted RIPA sample (5 µL RIPA sample and 95 µL PBS-EDTA) or standard (95 µL standard and 5 µL RIPA) was added to 200 µL of DMMB solution, and the extinctions were determined spectrophotometrically at 595 nm. GAG content was determined using the generated standard curve corrected for DNA content and expressed as µg GAG/ng DNA. DNA content in the same RIPA samples was determined using SYBR^®^ Green I Nucleic Acid stain (Invitrogen). A serially diluted standard curve of genomic control DNA (DNA Ladder G571A, Promega) in TE buffer was included to quantify DNA concentration in the samples. Before measurement, RIPA samples were diluted in TE buffer (1 µL RIPA sample and 99 µL TE buffer) and standard were prepared (99 µL standard in TE and 1 µL RIPA buffer). SYBR^®^ Green was diluted 10,000 times in TE buffer, and 100 µL of this solution was added to 100 µL of the above prepared samples or standards. Fluorescence was determined in standard 96-well plates in a SpectraFluor Plus reader (Tecan): excitation 485 nm and emission 535 nm. DNA concentration was determined using the standard curve.

### Gene Expression Analysis

Total RNA was extracted from dedifferentiated chondrocytes before encapsulation procedure, encapsulated chondrocytes cultured in alginate-based scaffolds, or the same cells maintained in micromass by using the RNeasy Mini Kit (Qiagen, Hilden, Germany), according to the manufacturer’s instruction. Total RNA was used for reverse transcription and stored at −80°C. Briefly, cDNA was synthesized from total RNA (500 ng) in a 20-µL reaction volume using the TaqMan High-Capacity cDNA Reverse Transcription kit (Applied Biosystems), as previously described (Lolli et al., 2014).

Gene expression analyses by RT-qPCR were performed for Col1A1, Col2A1, Aggrecan, and Col10A1 mRNA levels. The following Taqman probes were used: Col1A1 5′FAM-AAGACGAAGACATCCCACCAATCAC-NFQ3′, Col2A1 5′FAM-CCTGGTCTTGGTGGAAACTTTGCTG-NFQ3′, Aggrecan 5′FAM-CGCTGCCAGGGATCCTTCCTACTTG-NFQ3′, Col10A1 5′FAM-ATAAAGAGTAAAGGTATAGCAGTAA-NFQ3′ (Life Technologies, Carlsbad, CA, USA).

The CFX96™ PCR detection system (Bio-Rad, Hercules, CA, USA) was used, and results were calculated using the 2^−ΔΔCt^ method using glyceraldehyde 3-phosphate dehydrogenase as reference gene for normalization.

### Alcian Blue Staining

Glycosaminoglycan content was assessed by Alcian Blue staining in monolayered cells. Cells were rinsed with PBS and fixed in 10% formaldehyde in PBS for 10 min. Cultures were then stained with Alcian Blue pH 2 (1% in 3% acetic acid) (Sigma-Aldrich Chemical Co., St. Louis, MO, USA) 30 min at 37°C. Subsequently, cells were washed with water and observed using a Leitz microscope. The presence of GAG deposits appeared as blue staining areas.

### Immunocytochemistry

Immunocytochemistry analysis was performed on freshly, dedifferentiated, and recovered chondrocytes employing the ImmPRESS (Vectorlabs, Burlingame, CA, USA) or 4plus AP universal (Biocare Medical, Concord, CA, USA) detection kits. Cells grown in chamber slides were fixed in cold 100% methanol and permeabilized with 0.2% (v/v) Triton X-100 (Sigma-Aldrich Chemical Co., St. Louis, MO, USA) in TBS (Tris-buffered saline). Cells were treated with 3% H_2_O_2_ in TBS and incubated in 2% normal horse serum (Vectorlabs, Burlingame, CA, USA) for 15 min at room temperature. After the incubation in blocking serum, the different primary antibodies were added and incubated at 4°C overnight: polyclonal antibodies for Col1A1 (rabbit anti-human, 1:200 dilution, Santa Cruz Biotechnology, CA, USA), Col2A1 (mouse anti-human, 1:200 dilution, Abcam, Cambridge, UK), Aggrecan (mouse anti-human, 1:200 dilution, Santa Cruz Biotechnology, CA, USA), and Sox9 (rabbit anti-human, Santa Cruz Biotechnology, CA, USA). Cells were then incubated in Vecstain ABC (Vectorlabs, Burlingame, CA, USA) or Universal AP detection (Biocare Medical, Concord, CA, USA) reagents for 30 min and stained, respectively, with DAB solution (Vectorlabs, Burlingame, CA, USA) or Vulcan Fast Red chromogen kit (Biocare Medical, Concord, CA, USA). After washing, cells were mounted in glycerol/TBS 9:1 and observed using a Leitz microscope. Quantitative image analysis of immunostained cells was obtained by a computerized video-camera-based image-analysis system (with NIH, USA ImageJ software, public domain available at: http://rsb.info.nih.gov/nih-image/) under brightfield microscopy. Briefly, images were grabbed with single stain, without carrying out nuclear counterstaining with hematoxylin, and unaltered TIFF images were digitized and converted to black and white picture to evaluate the distribution of relative gray values (i.e., number of pixels in the image as a function of gray value 0–256), which reflected chromogen stain intensity. Images were then segmented using a consistent arbitrary threshold 50% to avoid a floor or ceiling effect and binarized (black versus white); total black pixels per field were counted, and average values were calculated for each sample. Three replicate samples and at least four fields per replicate were subjected to densitometric analysis. We performed the quantification of pixels per 100 cells and not per area in order to take into account the different cell morphology and confluence.

### Transmission Electronic Microscope (TEM) Analysis and Toluidine Staining

Cell-containing microfibers were fixed in glutaraldehyde 2.5% buffered solution and osmium tetroxide 2% buffered solution and dehydrated through an ethanol gradient; samples were araldite embedded (ACM Fluka Sigma-Aldrich Co., St. Louis, MO, USA) and the ultra-thin sections of a selected area were contrasted with uranyl acetate lead citrate and observed at transmission electron microscopy (TEM; ZEISS EM 910 electron microscope; Zeiss, Oberkochen, Germany). For toluidine staining, 5 µm sections from the same specimens were obtained with a glass blade. Sections were stained with toluidine blue, mounted in glycerol, and observed using a Leitz microscope.

### Microfibers Cryopreservation

Cryopreservation of A*f*, AG*f*, and AUBM*f* embedded chondrocytes was performed by adapting a previously published protocol (Pravdyuk et al., 2013). Microfibers embedded cells were directly freezed in standard culture medium with 10% FCS and supplemented with 10% of DMSO. The samples were cryopreserved at −20°C for 30 min, maintained at −80°C for 24 h, and then immersed in liquid nitrogen, where they were kept until the thawing day. Cryovials within microfibers were thawed in a warm water bath (37°C) and, when ice was totally melted, microfibers were washed twice with culture medium and subsequently cultured in standard conditions prior to performing morphological, viability, and immunocytochemical analysis.

### Statistical Analysis

All cell-related experiments were repeated with chondrocytes derived from five different donors and performed in triplicate for each donor. Data are presented as means ± SEM. The normal distribution of data was verified using the Kolmogorov–Smirnov test. In case of single comparison, statistical significance was determined by paired Student’s *t*-test for normally distributed data and Wilcoxon matched-pairs signed-ranks test for non-normally distributed data. In case of multiple comparisons, statistical significance was analyzed by one-way analysis of variance (ANOVA) and Bonferroni *post hoc* test if the values followed a normal distribution, or by Kruskal–Wallis analysis (non-parametric one-way ANOVA) and Dunn’s *post hoc* test if the values were not normally distributed. For all statistical analyses, differences were considered statistically significant for *p*-values ≤0.05.

## Results

### Production and Characterization of Composite Microfibers

**Figure 2:**
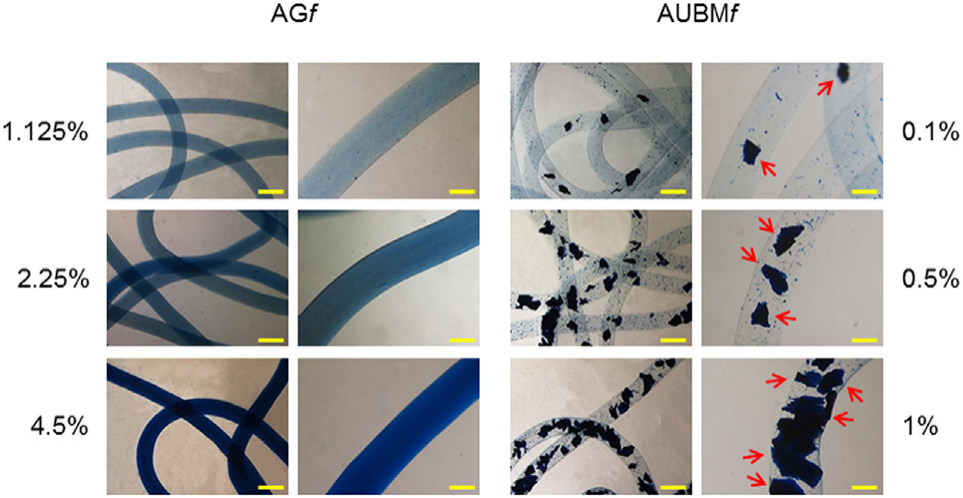
Distribution of gelatin and UBM in composite microfibers. Different amounts of gelatin (1.125, 2.25, and 4.5% w/v) or UBM (0.1, 0.5, and 1% w/v) were added to the alginate solution, respectively. Microfibers containing different amount gelatin/UBM were stained with Coomassie Blue Brilliant. The presence and distribution of UBM particles are indicated by arrows. Bar corresponds to 1 mm in lower magnification photomicrographs and to 400 µm in higher magnification images.

Alginate (A*f*), alginate-gelatin (AG*f*), and alginate-UBM (AUBM*f*) microfibers were produced by a microfluidic procedure by a snake micromixing chip, consisting in two inlets, a mixing element, and an outlet tube with an internal diameter of 700 µm (Figure 1A). The dimensional analysis of photomicrographs of microfibers produced at different flow rates (from 0.5 to 1.5 mL/min) demonstrates that the microfluidic procedure allows a good control on microfibers’ morphology and dimensions (Figures 1B,C). Microfibers present a smooth surface and a uniform morphology, with constant dimensions (in diameter); particularly, the pumping rate has an important effect of the microfiber dimensions. High pumping rates resulted in an increase in microfiber diameters; this behavior is indeed attributed to the well-known Barus effect (Malkin et al., 1976). Moreover, the analysis of the photomicrographs reported in Figures 1B,C demonstrates that the addition of gelatin or UBM does not affect the general microfiber morphology (i.e., in term of dimension and surface smoothness). Both gelatin and UBM caused only a moderate enlargement of the microfiber diameter. The produced microfiber presents good structural and morphological properties as proved by the Coomassie Blue Brilliant staining (Figure 2). Particularly, AG*f* are characterized by uniform staining, since gelatin is homogeneously present within the entire microfiber structure (i.e., being gelatin soluble in the aqueous sodium alginate); on the contrary, AUBM*f* display the presence of UBM particles evenly distributed within the microfiber matrix arrowed in Figure 2 (i.e., being UBM particles unsoluble in water). Therefore, the images reported in Figure 2 indicate that the snake micromixing chip allowed a homogeneous distribution of gelatin and ECM along the whole microfiber.

With respect to the concentrations of biomaterial used for the preparation of microfibers (sodium alginate, gelatin, and UBM), the selected amounts were chosen on the basis of a large number of previously published observations (Penolazzi et al., 2010, 2012; Mazzitelli et al., 2013; Angelozzi et al., 2015; Vecchiatini et al., 2015). For instance, alginate is typically used in the concentration range comprised between 0.5 and 2.5% (w/v) depending on the stiffness required by the specific application of the gel.

### Chondrocytes Expansion and Dedifferentiation Process

**Figure 3:**
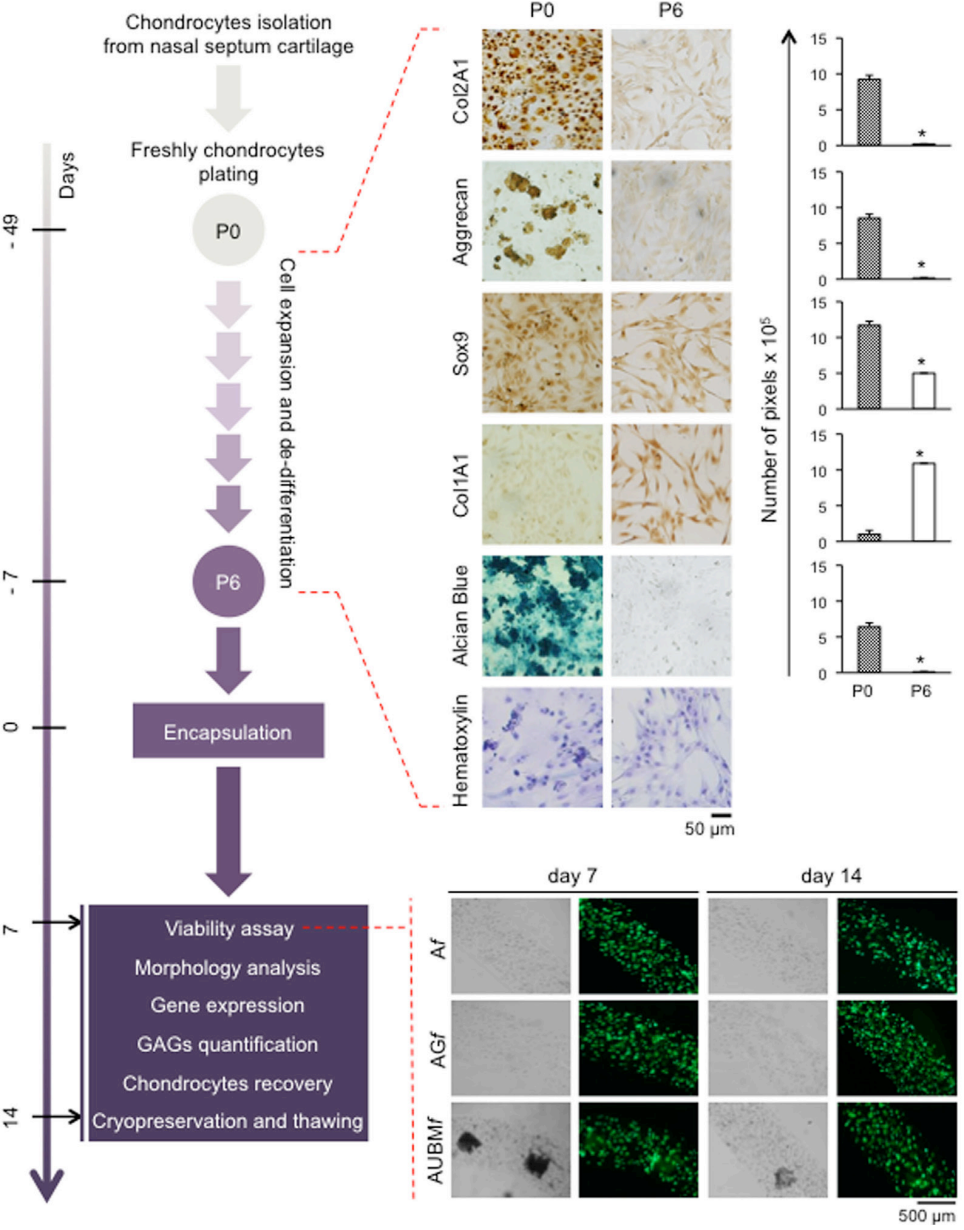
Schematic representation of the experimental approach. Human chondrocytes were isolated from nasal septum biopsy, expanded, and dedifferentiated up to the sixth culture passage (P6). Cells were then embedded in A*f*, AG*f*, and AUBM*f* and subjected to the indicated analysis at days 7 and 14. Dedifferentiation process from passage 0 (P0) to passage 6 (P6) has been monitored: protein expression of cartilage-related genes (Col2A1, Aggrecan, and Sox9), and Col1A1 was investigated by immunocytochemical analysis. Alcian Blue staining for sulfate glycosaminoglycans (GAGs) detection is also reported. Cell morphology was evaluated by hematoxylin staining. Representative optical photomicrographs are reported. Pictures of at least four random fields of three replicates were captured for densitometric analysis using ImageJ software. Data are presented as means of pixels per 100 cells ±SEM (*p ≤ 0.05). Cell viability of embedded cells has also been investigated, and optical and fluorescence photomicrographs after double staining with Calcein-AM/propidium iodide at days 7 and 14 of culture in basal medium are reported. The green fluorescence indicates the presence of calcein-labeled live cells, while propidium iodide-labeled dead cells are revealed by red fluorescence. Merged photomicrographs are reported.

To obtain dedifferentiated cells, P0 chondrocytes from five different donors were expanded up to six passages (Figure 3). As expected (Caron et al., 2012), the cartilaginous phenotype was progressively lost over several passages in culture. In fact, compared to P0 freshly isolated chondrocytes, P6 expanded chondrocytes exhibited a substantial change in cell morphology, from rounded to flattened fibroblast-like shape as it is evident from hematoxylin staining. Immunohistochemical analysis showed that expansion dramatically decreased the expression of typical chondrogenic markers including collagen type II (Col2A1), aggrecan, Sox9, and, in contrast, increased the positivity for collagen type I (Col1A1). Dedifferentiation status was confirmed by a dramatic decrease of sulfate GAG content in ECM composition (as demonstrated by Alcian Blue staining).

P6 chondrocytes expanded as dedifferentiated chondrocytes were then embedded in composite microfibers, which were subjected to specific analyses at days 7 and 14, as described below.

### Embedding of Dedifferentiated Chondrocytes in Composite Microfibers

Dedifferentiated chondrocytes were suspended, at 2 × 10^6^ cells/mL in (i) alginate 2%, (ii) alginate 2% plus gelatin 2.25%, or (iii) alginate 2% plus UBM 0.5%. The different cells/biomaterial suspensions were used to produce microfibers (A*f*, AG*f*, and AUBM*f*) by letting flow the cell suspension in a BaCl_2_ gelling bath at a pumping rate of 1.5 mL/min, resulting in microfibers with diameters comprised between 650 and 750 µm. The obtained alginate-based microfibers were maintained in culture medium without chondrogenic inducers up to 14 days, and the embedded cells were analyzed for viability at different time of culture. As shown in the lower part of Figure 3, after embedding in microfibres, cell viability was not compromised as confirmed by double staining with Calcein-AM/PI.

**Figure 4:**
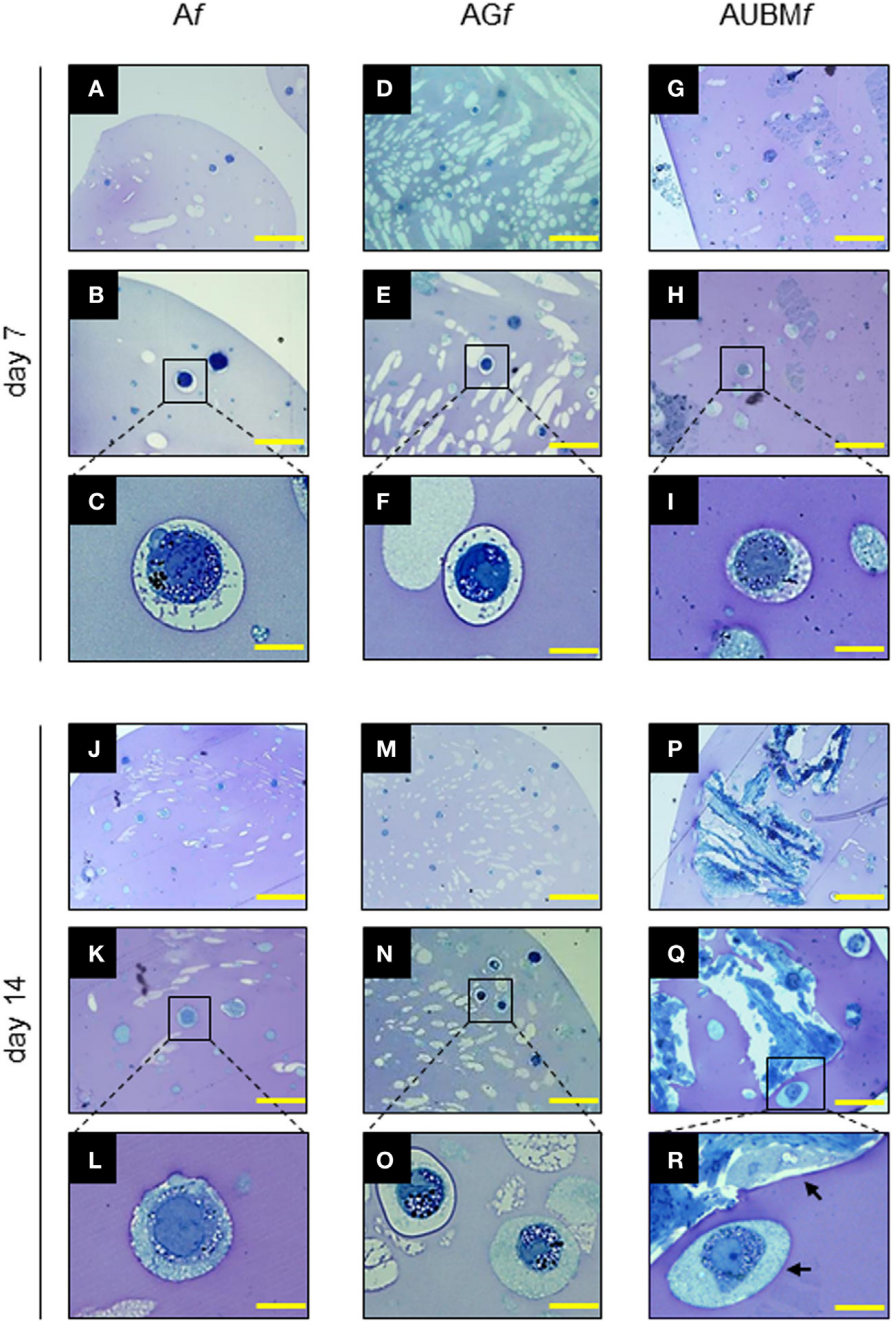
Properties of the embedded cells: presence of secretory vesicles and metachromatic areas in the extracellular matrix (ECM). A*f*, AG*f*, and AUBM*f* embedded cells at days 7 and 14 of culture were stained with toluidine blue. Representative photomicrographs showed the presence of ECM deposition in the pericellular space of the single cells (as clearly evidenced in the higher magnification images) and the presence of metachromatic area (pink). Black arrows: single or UBM-attached cell. Bar corresponds to 50 µm for photomicrographs **(A,D,G,J,M,P)**, to 25 µm for photomicrographs **(B,E,H,K,N,Q)**, and to 10 µm for photomicrographs **(C,F,I,L,O,R)**.

The properties of the embedded cells were further investigated in term of cell morphology, and proteoglycan deposition, by toluidine blue metachromasia of matrix. As shown in Figure 4, the embedded cells revert to a spherical shape in just 7 days. Interestingly, during the entire period of culture, up to 14 days, the embedded cells are mainly present as single cells, well dispersed in the alginate, showing an aspect resembling the environment of mature chondrocytes in cartilage. In addition, the presence of metachromatic areas with secretory vesicles in a well-defined pericellular space at days 7 and 14 was clearly detectable, indicating the effective release and deposition of cartilage-like ECM. The A*f*, AG*f*, and AUBM*f* composite microfibers represent therefore a microenvironment supporting the maturation of chondrocyte phenotype also in the absence of chondrogenic inducers.

### Chondrogenic Properties of Embedded Cells

**Figure 5:**
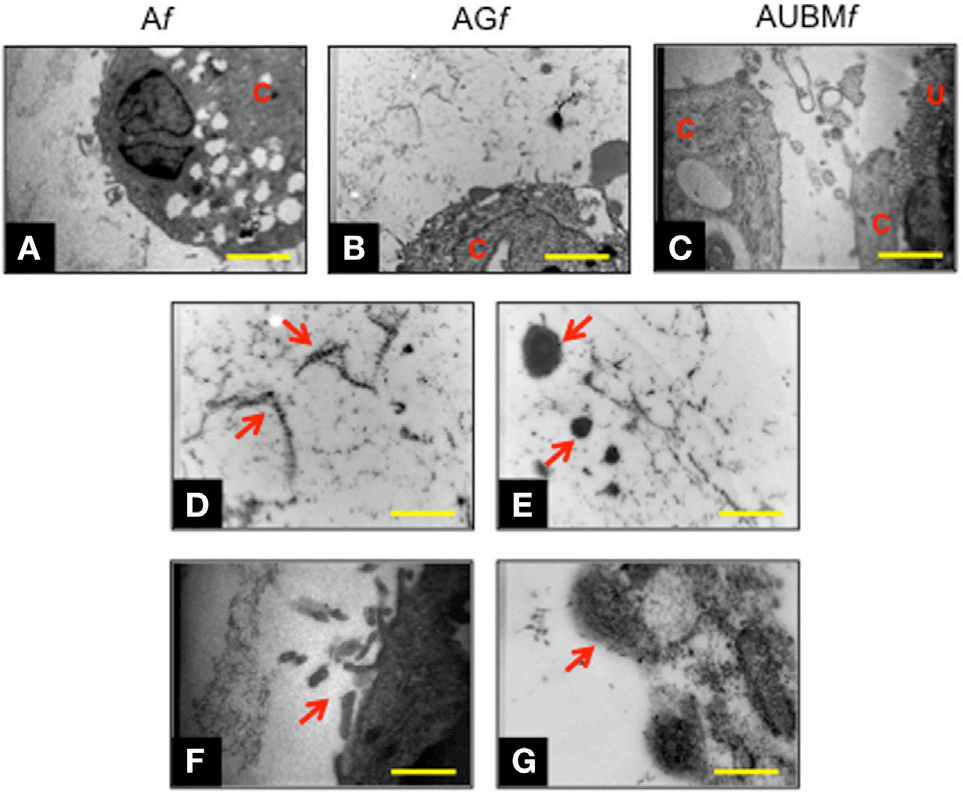
Chondrogenic properties of A*f*, AG*f*, and AUBM*f* embedded cells: transmission electronic microscope ultrastructural analysis at day 14 of culture. Lower magnification photomicrographs showed the ultrastructure of an embedded redifferentiated chondrocyte in A*f* **(A)**, AG*f* **(B)**, and AUBM*f* **(C)**. In the images at higher magnification, red arrows indicated the presence of ECM dense material and collagen fibers with their typical banding pattern in the pericellular space **(D,E)** and evidenced the ECM-containing vesicles release from embedded cells **(F,G)**. Bar corresponds to 2.6 µm in a-c, 1 µm in **(D–F)** and 0.4 µm in grams. C, cell; U, UBM.

Transmission electronic microscope ultrastructural analysis of embedded cells at day 14 revealed a high cellular secretory activity. As shown in Figure 5, the presence of secretory vesicles containing ECM dense materials, and collagen fibers with their typical banding pattern is clearly appreciable. The released materials accumulated and assembled in a sparse matrix in the surrounding lacuna, resembling the biological microenvironment of chondrocytes. These preliminary evidences confirmed the deposition of a cartilage-like ECM in this area, as hypothesized after the toluidine blue staining (see Figure 4). Interestingly, AUBM*f* showed the presence of cells able to interact with each other and with UBM flakes (characterized by typical collagen fibers) located in the closed areas.

**Figure 6:**
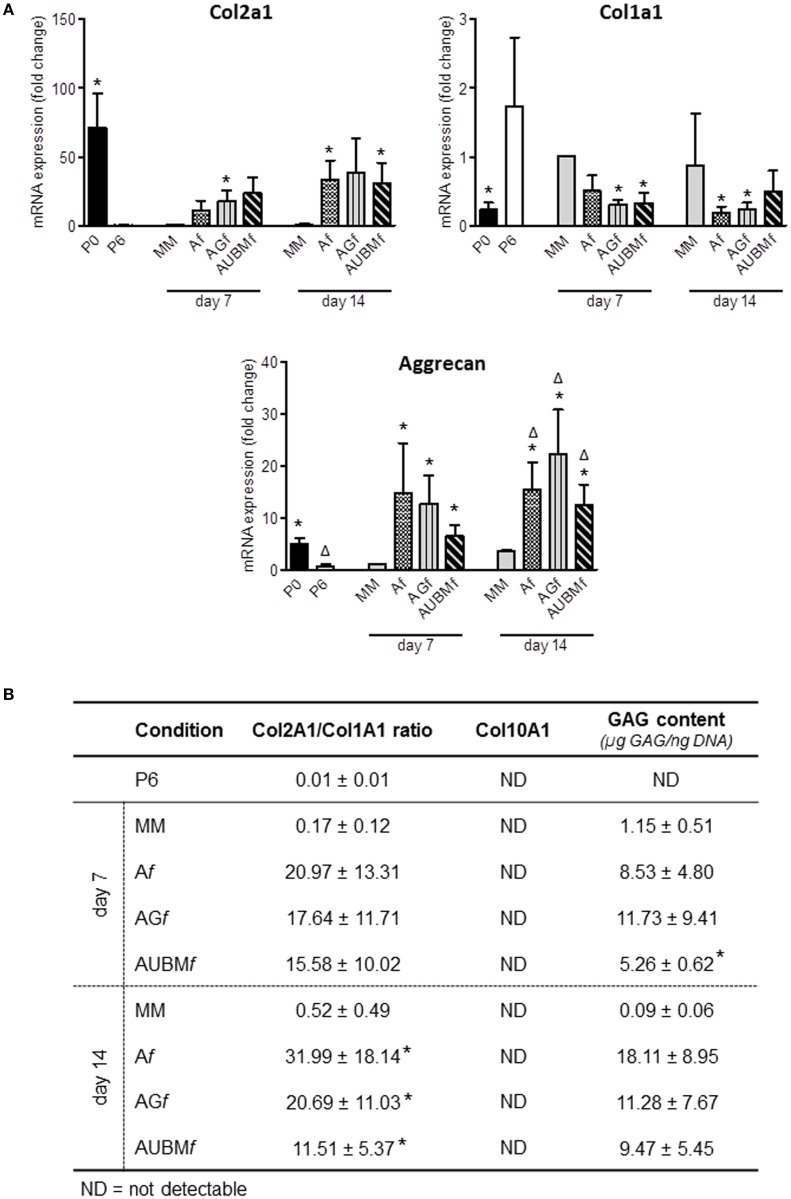
Chondrogenic properties of A*f*, AG*f*, and AUBM*f* embedded cells: analysis of cartilage-specific markers. A*f*, AG*f*, and AUBM*f* embedded redifferentiated chondrocytes or chondrocyte micromasses (MM) cultured for 7 or 14 days were compared for chondrogenic capacity by analyzing the expression of cartilage markers. **(A)** The expression of Col2A1, Col1A1, and aggrecan was evaluated by RT-qPCR. Values obtained from freshly isolated (P0) and dedifferentiated chondrocytes (P6) are also included. Results were calculated using 2^−ΔΔCt^ method, and data are presented as fold change means respect to MM day 7. **(B)** Quantification of Col2a1/Col1a1 ratio, Col10A1 expression, and GAG content for each experimental condition are reported. Col10a1 expression was assessed by RT-qPCR and resulted not detectable (ND) in all tested conditions. GAG content was quantified by DMMB staining on cellular lysates, and values are reported as µg GAG/ng DNA. All data are presented as means ± SEM of three independent experiments performed on chondrocytes from four different donors (*n* = 4). Statistical analysis was performed all conditions versus MM day 7 (*p < 0.05) or versus MM day 14 (^Δ^*p* < 0.05).

**Figure 7:**
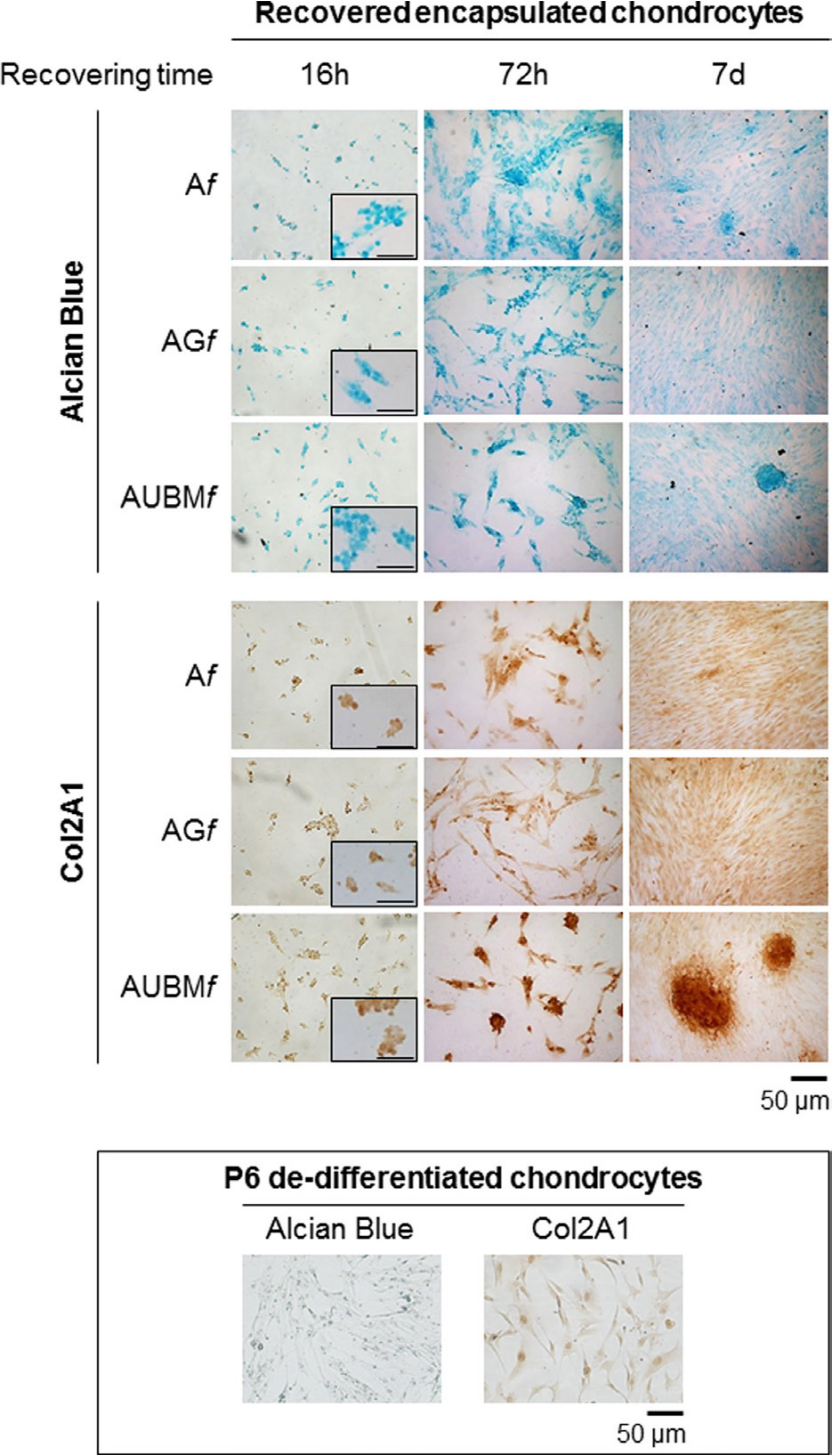
Behavior of the embedded cells after they are recovered from the microfibers. Redifferentiated chondrocytes were recovered from microfibers after 14 days of culture, reseeded, grown up to 7 days as monolayered culture in standard medium without adding chondrogenic inducers, and compared with P6 dedifferentiated chondrocytes. The cells were stained by Alcian blue for sulfated GAGs and immunostained for Col2A1 after growing in monolayer for 16 h, 72 h, or 7 days. Optical photomicrographs indicate the maintenance of acquired chondrogenic properties. Bar corresponds to 50 and 100 µm for the insets.

The chondrogenic properties of the embedded cells were then investigated with two different approaches (Figures 6 and 7). In a first step, embedded cells were harvested from microfibers at days 7 and 14 of culture and subjected to evaluation of chondrogenic markers. RT-qPCR revealed that the main cartilage-specific ECM component, Col2A1, and the major proteoglycan in cartilage, aggrecan, were highly expressed in cells embedded in A*f*, AG*f*, and AUBM*f* compared to chondrocytes cultured in standard micromass (MM) and P6 dedifferentiated chondrocytes (Figure 6A). Micromass culture system has been chosen instead of pellet culture since it may be maintained in culture medium also without adding TGFβ supplementation. The remarkable increase of Col2A1 expression and decrease of Col1A1 was particularly appreciable calculating the ratio between Col2A1 and Col1A1 (differential index) which significantly increased from 20 to 120 times in all embedded cells compared to MM cultured cells (Figure 6B). Moreover, gene expression analysis showed also the absence of expression of Col10A1, which encodes the collagen type 10 alpha 1 chain traditionally associated with chondrocyte hypertrophy, both at 7 and 14 days (Figure 6B). This suggested that microfiber environment is suitable to prevent undesired hypertrophic maturation that can widely affect the successful outcome of the graft transplantation. The reacquistiton of chondrogenic properties by the embedded cells was confirmed by GAG quantification performed as total GAG content normalized to DNA content (Figure 6B), and comparable GAG content was found in A*f*, AG*f*, and AUBM*f* embedded cells.

It is important to underline that with these experiments we also demonstrated that the small amount of TGFβ in the FCS containing medium is not sufficient to support chondrogenesis in standard conditions of MM that requires exogenous chondrogenic inducers. Conversely, microenvironments created by microfibers support redifferentiation capacity of expanded chondrocytes without the need of adding chondrogenic inducers.

In a second step, embedded cells were recovered at day 14 from the microfibers, reseeded, and grown up to 7 days as monolayered culture in standard medium without adding chondrogenic inducers. These experiments were aimed both at validating the previous RT-qPCR data by immunocytochemical analysis and at further demonstrating the intrinsic potency of microfibers in maintaining chondrogenic activity of the cells once released from the confined 3D microenvironment of the scaffolds.

Interestingly, even if plated in unfavorable conditions, the cells maintained a round morphology, a low adhesion ability, and generated microaggregates, demonstrating a behavior comparable to freshly isolated chondrocytes. As shown in Figure 7, the cells were intensively stained by Alcian blue for sulfated GAGs and immunostained for Col2A1. The presence of UBM was particularly effective in promoting the maintenance of chondrogenic phenotype as evidenced by a larger number of Col2A1 positive spontaneous microaggregates that persisted over 7 days.

### Cryopreservation of *Af, AGf*, and *AUBMf* Embedded Cells

**Figure 8:**
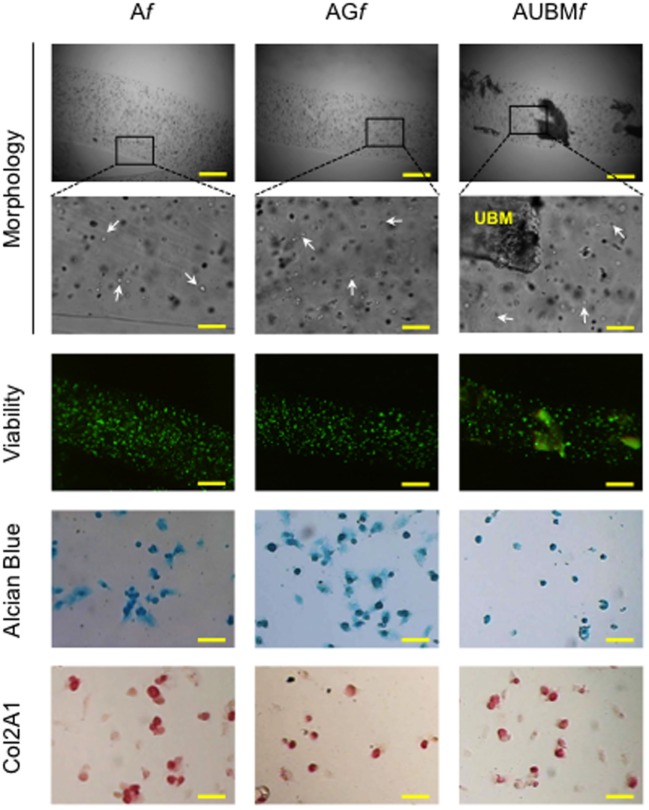
Cryopreservation of *Af, AGf*, and *AUBMf* embedded cells. A*f*, AG*f*, and AUBM*f* embedded redifferentiated chondrocytes were frozen, stored in liquid nitrogen, thawed, and assessed for cell morphology, viability, and chondrogenic properties. Optical images of microfibers show the rounded shape retention by the thawed embedded cells (particularly evident at higher magnification: see white arrows). Fluorescence merged images of the Calcein-AM (green)/propidium iodide (red) double staining demonstrated the high cell viability. The presence of GAGs and Col2A1 was detected by Alcian Blue staining and immunocytochemical analysis on the thawed embedded cells recovered from microfibers and grown in monolayer for 24 h. Bar corresponds to: 300 µm in lower magnification images of morphology and viability, 60 µm in higher magnification, and 50 µm in Alcian Blue staining and Col2A1 immunocytochemical analysis.

To explore the possibility to set up a bank of “microfibrous scaffolds embedded chondrocytes” for further *in vitro* and *in vivo* manipulations, we performed a preliminary assessment of the properties of thawed A*f*, AG*f*, and AUBM*f* embedded cells after freezing procedure. The redifferentiated chondrocytes were frozen directly within microfibers in complete culture medium supplemented with 10% DMSO, stored at −196°C in liquid nitrogen, thawed, and then assessed for cellular viability and chondrogenic properties. Similar to the freshly A*f*, AG*f*, and AUBM*f* embedded cells, thawed samples maintained highly viable cells with a round shaped morphology (Figure 8). When recovered from microfibers and grown in monolayer in standard medium without chondrogenic inducers for 24 h (the conditions are the same described in Figure 7), thawed samples preserved GAG production and Col2A1 expression as shown by Alcian Blue staining and immunocytochemistry (Figure 8).

Therefore, the alginate-based microfibers appear resistant to freezing and allow us to recover highly viable and functional cells also after thawing.

## Discussion

This paper describes the production of microfiber constructs containing redifferentiated chondrocytes into alginate hydrogels for the treatment of cartilage defects.

Cells have been embedded in hydrogels combined with ECM-derived components (i.e., gelatin or decellularized UBM) without any chondrogenic growth factors supplementation (i.e., TGFβ). Alginate was chosen as main constituent of the construct since its beneficial on improving chondrocyte properties and supporting chondrogenesis have been previously described (Moutos and Guilak, 2008). In addition, it has been demonstrated that the concomitant presence of ECM-derived materials can ameliorate the performances of the scaffold for cartilage TE, accelerating the tissue regeneration (Zhang et al., 2016).

The novelty of our experimental approach lies indeed not only in the microfluidic approach developed for microfibers production but primarily on the combined presence of alginate and gelatin/UBM in a multifunctional scaffold containing nasal chondrocytes. Moreover, the behavior of embedded cells has been investigated with various techniques, allowing to finely assess how the biomaterial constructs were mimicking cartilage physiological microenvironment.

It is important to underline that many researchers involved in cell-based regenerative medicine are promoting the employment of cells by ethical and non-invasive procedures such as those isolated from tissues representing surgical wastes. In this regard, nasal septum, easily harvested from surgical procedures with minimal morbidity, represents an ideal source for cartilage cells.

In this respect, the presented data, strengthen the interest toward chondrocytes from the nasal septum, which show superior and more reproducible chondrogenic capacity, compared with chondrocytes from articular cartilage (Kafienah et al., 2002; Rotter et al., 2002; Wolf et al., 2008; Mumme et al., 2016). The comparison with articular chondrocytes demonstrated that nasal chondrocytes were able to support the production of a cartilage matrix with adequate functional and biomechanical characteristics both *in vitro* and *in vivo* (do Amaral et al., 2012; Pleumeekers et al., 2014). Therefore, it is reasonable to suppose that good mechanical integrity and structural stability of surgical specimens from nasal septum may remain in the memory of chondrocytes if cultured in a favorable environment. This might explain the highly chondrogenic properties exhibited by our redifferentiated chondrocytes recovered from microfibers after long-term culture.

Importantly, the high passage (P6) cells were highly capable to redifferentiate when properly embedded in alginate-based microfibrous scaffolds. This finding is of particular relevance since P6 dedifferentiated chondrocytes are widely described as irreversibly dedifferentiated chondrocytes.

They are usually not recommended for transplantation since become apoptotic, inhibit key signaling proteins in the MAP kinase pathway, produce matrix-degrading enzymes, losing, as a whole, their chondrogenic potential definitively (Dell’Accio et al., 2001; Schulze-Tanzil et al., 2004). On the contrary, the alginate-based composite scaffolds allowed to support chondrogenic process of advanced dedifferentiated chondrocytes from monolayer passage P6. This evidence appear particularly notable since the use of cells at higher culture passages allows to expand the cell populations to a much higher level for further clinical applications, overcoming the issue of relative small and insufficient donor samples (Melero-Martin and Al-Rubeai, 2007).

Interestingly, the effects of 3D microenvironments based on alginate plus gelatin or UBM on the embedded cells occurred without the addition of the typical chondrogenic inducers such as TGFβ. This evidence represents an important added value considering the recently reported controversial action of TGFβ. Despite many well-described advantages, the use of differentiating agents is indeed questioned for the undesired off target effects and controversial outcomes. For instance, it has been reported that TGFβ may be detrimental for the redifferentiation process of chondrocytes and may promote the rapid and undesirable differentiation into fibroblast-like cells (Mueller et al., 2010; Narcisi et al., 2012). The negative effect of TGFβ in wound repair of cartilage has been also observed in several experimental models supporting the hypothesis that the inhibition of TGFβ may induce cartilage repair (Blaney Davidson et al., 2007; Khaghani et al., 2012) without the onset of undesired hypertrophy. Hypertrophy represents, in fact, one of the major drawbacks in autologous chondrocytes implantation and in MSC-based strategies (Melero-Martin and Al-Rubeai, 2007; Niethammer et al., 2014).

Notably, the here described composite microfibers represent a favorable microenvironment for the embedded cells supporting their viability and maturation. The alginate 3D matrix in combination with UBM prevents cell-to-cell contacts, mimicking the physiological microenvironment typical of mature cartilage. The embedded cells were indeed mainly present as single cells, well dispersed in the alginate, showing a morphology resembling that of mature chondrocytes in cartilage. This evidence is essential in view of the *in vivo* use of microfibers, showing for the first time that the presence of UBM is not detrimental for chondrocytes activity, rather it seems particularly effective in maintaining chondrogenic activity of the cells and their ability to form aggregates once released from the confined 3D microenvironment of the scaffolds (Figure 7). This point is intriguing and allow us to hypothesize that UBM may act as a guide for a proper cell arrangement when implanted within a complex mixture of structural and bioactive molecules such as cartilage tissue, without risk of rejection since decellularization removes or masks antigenic epitopes (Turner and Badylak, 2015). Moreover, considering that the use of decellularized allografts and xenografts in the repair of damaged cartilage is just beginning, our data may give useful information for generating biomimetic engineered cartilage constructs.

A further advantage of alginate microfibers relies on the cryoconservation of the cells for further therapeutic uses. Alginate microfibers appear resistant to freezing and allow to maintain highly viable and functional cells after thawing. Consequently, the presented scaffolds may be proposed as tool for *in vitro* redifferentiation process and recovering of an effective and functional chondrocyte population potentially able to produce a neocartilage tissue *in vivo*. This evidence is also important in view of a future chondrocyte bank that would be of great help as a permanent source of cartilage cells.

In conclusion, to the best of our knowledge, few reports focused on the effect of ECM-derived biomaterials in cartilage regeneration (Jin et al., 2007; Yang et al., 2008; Baugé et al., 2014; Grogan et al., 2014; Lee et al., 2014; Youngstrom et al., 2015) and in any case the combination with alginate properties has not been described. This interaction is an essential feature for the success of a TE strategy, allowing to maintain the low friction and the load-bearing characteristics of the native cartilage (Moutos and Guilak, 2008; Grogan et al., 2014). Therefore, the hypothesis to guide cartilage neoformation *in vivo* by such cell-based microfibrous constructs is worth further consideration, especially given that fibrous versus non-fibrous scaffolds offer interesting advantages for cell delivery in biomedical application since guided growth, alignment, and migration of cells are favored (Blaney Davidson et al., 2007; Yang et al., 2008; Grogan et al., 2014; Youngstrom et al., 2015). In conclusion, our results provide a proof of concept for developing a next experimental design based on the implantation of microfibers or recovered redifferentiated chondrocytes on animal models with critical size defect affecting the whole joint.

## Ethics Statement

Cartilage fragments from nasal septum were obtained from 15 donors between 25 and 60 years old, which underwent septoplasty surgery procedures, after informed consent and approval of the Ethics Committee of the University of Ferrara and S. Anna Hospital.

## Author Contributions

MA conception and design, collection and assembly of data, data analysis and interpretation, manuscript writing, and final approval of manuscript. LP, SM, EL, and AL conception and design, data analysis and interpretation, and final approval of manuscript. RP and CN conception and design, provision of study material, data analysis and interpretation, manuscript writing, and final approval of manuscript.

## Conflict of Interest Statement

The authors declare that the research was conducted in the absence of any commercial or financial relationships that could be construed as a potential conflict of interest. The reviewer, AB, and handling editor declared their shared affiliation, and the handling editor states that the process nevertheless met the standards of a fair and objective review.
