# Supplementary figures and images for: Dedifferentiated Chondrocytes in Composite Microfibers As Tool for Cartilage Repair
Source: Front Bioeng Biotechnol. 2017 Jun 13;5:35. doi: 10.3389/fbioe.2017.00035 (PMC5468460; doi:10.3389/fbioe.2017.00035)

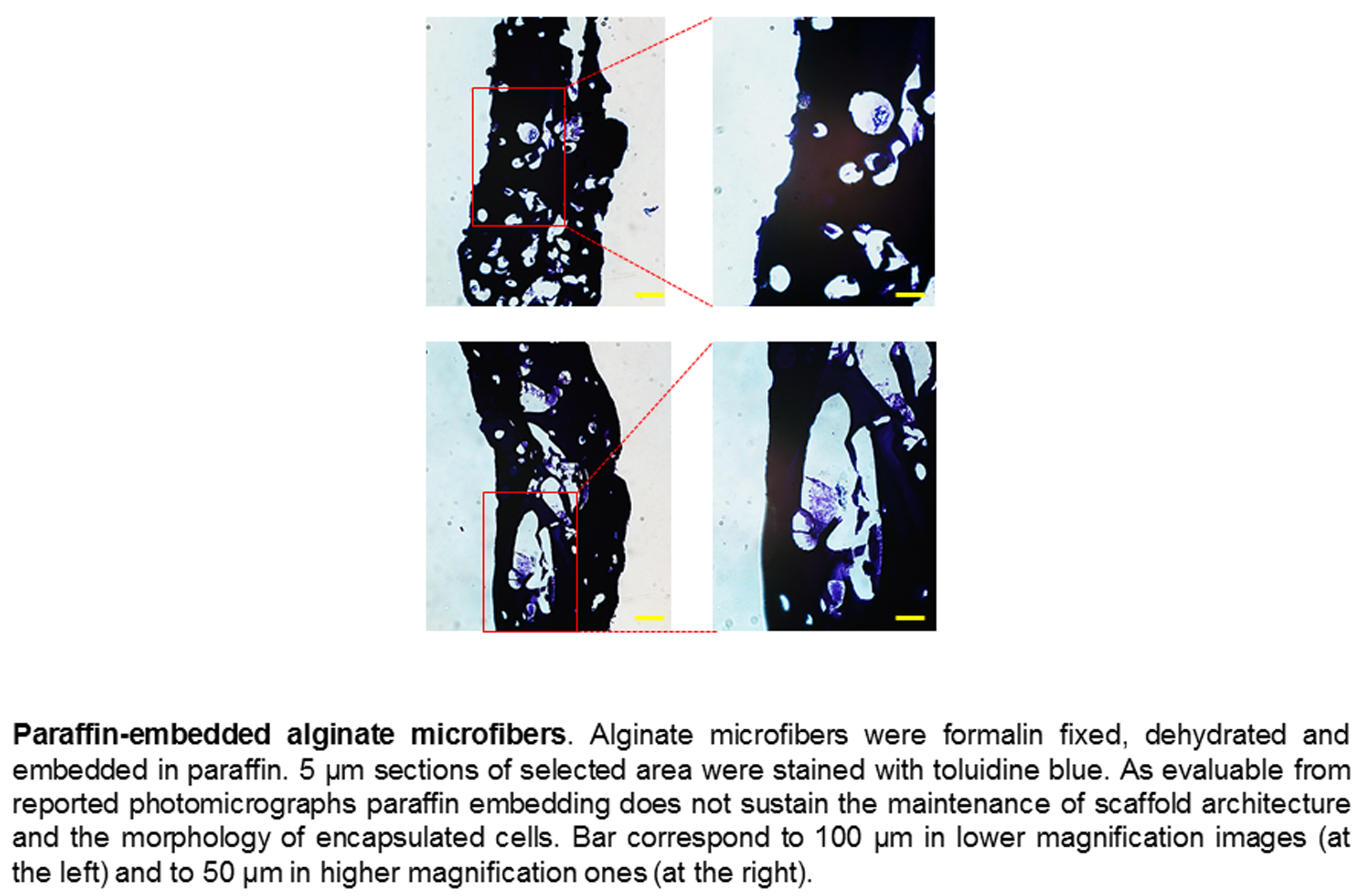

Supplement: Supplementary file 1 [file Image_1.TIF]
